# Minimal invasive percutaneous plate osteosynthesis (MIPPO) through deltoid-pectoralis approach for the treatment of elderly proximal humeral fractures

**DOI:** 10.1186/s12891-017-1538-9

**Published:** 2017-05-12

**Authors:** Li Zhao, Peng Yang, Lei Zhu, Ai-min Chen

**Affiliations:** Department of Orthopedic Trauma Surgery, Changzheng Hospital, the Second Military Medical University, 415 Feng Yang Rd., Huang Pu district, Shanghai, 200003 China

**Keywords:** Minimal invasive percutaneous plate osteosynthesis (MIPPO), Deltoid-pectoralis approach, Proximal humeral fractures

## Abstract

**Background:**

Current treatments for proximal humeral fractures include conservative treatment, conventional open reduction internal fixation (ORIF) and MIPPO through deltoid-splitting approach. The aim of this study was to evaluate the clinical outcome of MIPPO versus ORIF via the deltoid-pectoralis approach in elderly patients with proximal humeral fractures.

**Methods:**

Thirty-six patients with proximal humeral fractures were enrolled in this study. Following the randomized block and single-blinded principle, the patients were assigned to two groups and treated with either conventional ORIF or MIPPO, both through the deltoid-pectoralis approach. Surgical outcomes were evaluated by the NEER score, Constant-Murley score, blood loss, length of operation, radiological imaging and clinical examination. The patients were followed up for 4–24 (mean 10) months.

**Results:**

According to Constant-Murley score, the surgical outcome was excellent in 14 cases, satisfactory in 2 cases and unsatisfactory in one case in MIPPO group versus 10, 5 and 4 in conventional ORIF group. MIPPO was significantly advantageous over conventional ORIF in terms of NEER score, Constant-Murley, length of operation and intraoperative blood loss. In addition, MIPPO was also more advantageous in several indexes in patients with BMI > 26.0 and NEER type III fracture.

**Conclusion:**

The results of our study have demonstrated that MIPPO through the deltoid-pectoralis approach is an effective alternative for the treatment of proximal humeral fractures in elderly patients.

**Trial registration:**

The trial registration number (TRN): ChiCTR-INR-17011098 (retrospectively registered at 2017-04-09)

## Background

With the aging of society, osteoporosis-related fracture and its comorbidities including pneumonia, deep vein thrombosis (DVT), limb dysfunction, nerve injury and decubitus in elderly people have increasingly become major medical concerns in China [1, 2]. Proximal humeral fracture (PHF), which consists of 5% of all fractures, increased by more than 3 fold between 1970 and 2002, and about 70% of all 3/4-part PHF were seen in patients over 60 years [3–5]. Among conservative treatment, open reduction internal fixation (ORIF), minimal invasive percutaneous plate osteosynthesis (MIPPO), intramedullary nailing and arthroplasty reported in literature [6–14], which is the optimal treatment for PHF remains controversial. Due to poor bone quality, complications such as anemia, infection and delayed union are more common in elderly patients [15]. Conventional surgical methods of ORIF include the lateral deltoid approach and the deltoid-pectoralis approach. However, the lateral deltoid approach using the MIPPO technique was recently reported to associated with a risk of damage to blood supply of the deltoid and axillary nerve [4, 11, 15–28]. Compared with this approach, the deltoid-pectoralis approach requires extensive soft tissue reduction and may damage the anterior circumflex humeral artery and cephalic vein [16].

To provide an alternative option for the treatment of PHF in elderly patients, we for the first time used the MIPPO technique through the deltoid-pectoralis approach with the proximal humeral internal locking system to treat elderly PHF. The aim of the present study was to verify the advantages of the MIPPO technique through the deltoid-pectoralis approach by comparing the clinical outcomes of 17 cases treated with this technique and 19 cases treated with conventional ORIF through the deltoid-pectoralis approach in terms of NEER/Constant-Murley Score, intraoperative blood loss, length of operation and union time.

## Methods

Included in this study were 36 patients who attended our department for PHF between August 2014 and June 2016. The inclusion criteria included: (1) patients with freshly diagnosed PHF (NEER II/III); (2) patients with surgical indications; and 3) patients older than 55 years. Patients were excluded from the study if they: (1) had severe systemic diseases; (2) pathological fractures; and (3) primary neurovascular damage.

**Fig. 1:**
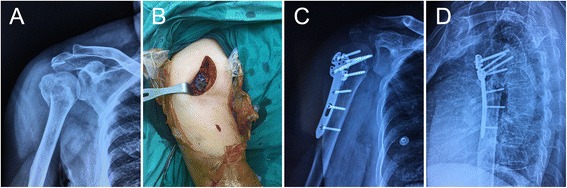
Follow-up data of a 66-year-old female. **a** Pre-operative X-ray shows an unstable right proximal humeral fracture. **b** intraoperative incisions (a 5-6 cm incision and a 1 cm incision). **c**/**d** Post-operative X-rays in in anteroposterior and lateral views show a good reduction and proper placement of the plate

All the 36 patients in this prospective study were diagnosed as unilateral PHF and individually divided into MIPPO (*n* = 17) and ORIF (*n* = 19) groups with the principle of randomized block. All fractures were classified according to NEER classification based on X-ray and CT presentations (Fig. 1a). There were 15 cases of NEER II PHF and 21 cases of NEER III PHF (Table 1).

**Table 1:** Demographics of the patients

Characteristic		Value	MIPPO group	ORIF group	*P* value
Gender	M/F	21/15	9/8	12/7	0.548
Age	Average±SD	64.0±5.8	64.3±6.7	63.6±5.0	0.759
BMI(kg/m^2^)	Average	25.9±3.0	26.8±3.2	25.1±2.5	0.08
Mechanism	Traffic accident	7	3	4	
	Fall	17	7	10	
	Sports	12	7	5	
NEER classification	II	15	8	7	
	III	21	9	12	
time between injury and operation(days)	Average±SD	2.7±0.8	2.8±0.7	2.7±0.9	
Follow-up(months)	Average(range)	10(4-24)			

On admission, all the patients received routine treatments including hemostasis, detumescence, analgesia, temporary fixation and blood/imaging examinations. The mean time from injury to operation was 2.7 days. In MIPPO group, the patient was laid in a beach position and an approximately 5 cm incision was made along the coracoid process of the scapula below the pectoral-deltoid clearance under general anesthesia. The cephalic vein was then exposed and protected with caution. After properly isolating the soft tissue and sternoclavicular fascia, the humeral head was exposed. A 2-cm skin incision was made longitudinally underneath the proximal incision. The bone block was reduced and provisionally fixed by Kirschner wires as confirmed by fluoroscopy. Then, a subcutaneous tunnel was made from both incisions to the fracture site over the periosteum deep to the deltoid muscle and an ITS proximal humeral locking plate (GE medical, USA) was inserted from the proximal incision and adjusted to a suitable height. A screw was fixed at both proximal and distal ends of the plate separately. If the X-ray image proved that the position of the fracture end and the plate were acceptable, 4–5 screws were fixed proximally while 2–3 screws were fixed distally (Fig. 1b). Allograft bone was grafted if there existed bone loss. No allograft was grafted in all 36 patients of the present study. After checking surgical instruments and irrigation, the incisions were closed.

In conventional ORIF group, the patient was laid in the same position and received conventional surgery through the deltoid-pectoralis approach. An approximately 12-cm incision was made along the medial border of the deltoid muscle from the coracoid process of the scapula. After proper exposure of the soft tissue and muscle according to the fracture site, the fracture was carefully reduced and fixed by Kirschner wires. Similar to MIPPO group, an ITS proximal humeral locking plate (GE Medical, USA) was gently inserted and screws were fixed based on real time X-ray imaging. The length of operation and intraoperative blood loss were recorded during surgery. Two groups shared the same type of surgical instruments, plates and screws and were conducted by Prof. Chen AM.

Patients received routine postoperative treatments, and functional rehabilitation was initiated about 6 days after operation at the time of discharge. Elbow flexion to 90° and external rotation to 0° for 4 weeks was suggested to reduce the stretching force of the shoulder joint. Active exercise of the shoulder joint would begin 4 weeks postoperatively depending on the healing situation. Complications were defined as infections, nerve and vascular injuries, decubitus, pneumonia and nonunion. Nonunion was diagnosed when the fracture remained unhealed 9 months after operation and no evidence of healing was observed for subsequent 3 months. Follow-up visits were arranged monthly in the first 6 months, and then at 24 and 48 months postoperatively for clinical and radiographic examinations. The healing of fracture and complications were evaluated according to the anteroposterior and lateral views of radiography (Fig. 1c and d). Clinical outcomes were evaluated by NEER/Constant-Murley score expressed as mean ± SD. All 36 patients were able to complete the visual analogue scales (VAS) for pain on their own at the final follow-up. The VAS pain scale ranged from 0 (no pain) to 10 (severe pain), and patients estimated the mean pain level in the injured limb during the previous month (Table 2). The evaluations were accomplished at 6 months postoperatively or at the latest visit in patients who were discharged within 6 months. Statistical analysis was performed by SPSS13.0 (SPSS Inc., Chicago, IL). Comparisons between conventional ORIF group and MIPPO group were performed using the *t*-test, and *p* < 0.05 was considered statistically significant.

**Table 2:** Follow-up data of the patients

Characteristic		Value	MIPPO group	ORIF group	*P* value
Constant-Murley score	Average±SD	87.8±1.9	88.8±1.0	86.9±2.1	0.001
NEER score	Average±SD	86.5±2.2	87.4±1.2	85.7±2.6	0.019
Intraoperative blood loss(ML)	Average±SD	137.7±22.0	129.2±17.8	145.3±23.0	0.026
length of operation (minutes)	Average±SD	57.8±8.1	53.6±7.3	61.4±7.0	0.002
Union time(months)	Average±SD	4.5±1.0	4.5±1.1	4.5±1.0	0.873
VAS(Visual Analogue Score(0-10):indicates pain from minimum to maximum.)	Average±SD	3.1±1.3	3.1±1.3	3.2±1.3	0.726
The short form (36) health survey.	Average±SD	125.6±9.1	129.4±7.8	122.3±8.9	0.017
Complications			Incision infection(1);pneumonia(1)	Incision infection(2); decubitus(1)	

## Results

**Fig. 2:**
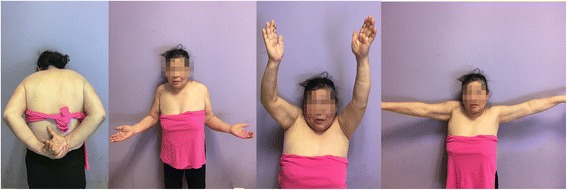
At the 6-month follow-up, the patient had a good shoulder function. The 6-month constant score was 90

Characteristics of the 36 patients and statistical results are displayed in Table 2. No nerve and vascular injury or nonunion was noticed in all the 36 patients. Complications such as incisional infection, pneumonia and decubitus were cured before the patients were discharged from the hospital. The indexes of NEER score, Constant-Murley score, length of operation and intraoperative blood loss in MIPPO group were better than those in ORIF group (*p* < 0.05). Meanwhile two groups showed no significant difference in the statistical results of VAS and union time. To determine correlations of the NEER type, BMI and surgical method with the therapeutic outcome, all patients were divided into subgroups according to the NEER type (II or III) (Table 3) or BMI (≥26.0 or <26.0) (Table 4) (Fig. 2).

**Table 3:** Demographics of statistical data of subgroup by NEER type

Characteristic	NEER type II			NEER type III		
	MIPPO	ORIF	*P* value	MIPPO	ORIF	*P* value
Constant-Murley score	88.4±0.7	87.1±2.7	0.231	89.2±1.0	86.8±1.8	**0.001**
NEER score	87.3±0.7	86.6±2.6	0.494	87.4±1.5	85.2±2.6	**0.030**
Intraoperative blood loss(ML)	131.0±12.3	148.3±21.1	0.070	127.7±22.2	143.6±24.8	0.145
length of operation (minutes)	55.3±7.9	59.9±6.5	0.245	52.2±6.8	62.3±7.4	**0.005**
Union time(months)	4.5±1.4	4.9±1.1	0.595	4.4±0.9	4.3±0.9	0.779
VAS	3.8±1.0	3.6±1.1	0.755	2.4±1.2	3.0±1.3	0.345
SF36	125.8±9.6	122.6±6.2	0.469	132.6±4.1	122.1±10.5	**0.011**

**Table 4:** Demographics of statistical data of subgroup by BMI index

Characteristic	BMI<26.0			BMI≥26.0		
	MIPPO	ORIF	*P* value	MIPPO	ORIF	*P* value
Constant-Murley score	89.1±0.69	86.7±2.31	**0.01**	88.6±1.1	87.1±1.9	**0.048**
NEER score	87.7±1.25	86.0±2.98	0.17	87.1±1.1	85.3±2.3	**0.043**
Intraoperative blood loss(ML)	118.1±16.3	135.2±16.5	0.052	137.0±14.9	156.6±24.9	0.052
length of operation (minutes)	51.4±7.3	60.4±8.2	**0.035**	55.2±7.2	62.6±5.7	**0.02**
Union time(months)	5.1±0.9	4.6±1.1	0.292	4.0±1.1	4.4±0.9	0.336
VAS	2.5±1.6	2.8±1.2	0.745	3.4±1.0	3.7±1.2	0.603
SF36	128.3±11.4	125±8.1	0.495	130.1±4.6	119.2±9.3	**0.004**

It was found that both NEER type II and III had favorable impact on intraoperative blood loss. In addition, Constant-Murley score, NEER score, length of operation and SF36 score were better in NEER type III patients of MIPPO group as compared with the conventional ORIF group (*P* < 0.05), suggesting that the prognosis in NEER type III patients may be better than that in NEER type III patients of the same MIPPO group (Table 3). Surprisingly, in patients with BMI < 26.0, there was no significant difference in Constant-Murley score, NEER score, intraoperative blood loss, length of operation and SF36 score between MIPPO and ORIF groups, while the difference was significant in patients with BMI > 26.0 (Table 4), suggesting that MIPPO technique may have better effects in over-weight individuals.

## Discussion

MIPPO through the deltoid-pectoralis approach seems superior to conventional ORIF through the deltoid-pectoralis approach in the treatment of PHF in elderly patients in terms of Constant-Murley score, NEER score, intraoperative blood loss, length of operation and SF36 score. The application of MIPPO in elderly patients can not only decrease intraoperative injury and complications but avoid damage to blood supply of the deltoid muscle and axillary nerve.

To explore possible factors influencing the application of MIPPO technique, we also included the NEER type and BMI into statistical analysis. As described above, the prognosis was relatively better in patients over 65 years or with NEER type III or BMI index > 26.0. It seems that NEER type III and over-weight patients who were likely to have a worse prognosis [14] may acquire a relatively better outcome though MIPPO versus conventional ORIF, especially in patients with more complex PHF or those with a poor general condition.

But we found no significant difference in union time between the two groups. Some previous studies [16, 17] reported that MIPPO may prolong the union time in patients with humeral shaft fractures. We think that one of the possible explanations is that compared with the proximal humerus, the humeral shaft receives less blood supply, and thus sufficient blood supply plays a bigger role in fracture union in humeral shaft fractures than that in PHF. Therefore, MIPPO offers a better effect on union time in humeral shaft fractures, knowing that it is able to decrease soft tissue and vascular injury and increase blood supply in fracture union. However, this hypothesis needs to be confirmed in more cases. Compared with the deltoid-splitting approach reported in previous studies [4, 11, 14, 17], we think that the damage to blood supply could be dimished by protecting the integrity of the deltoid muscle to help bone healing and avoid damage to the axillary nerve in MIPPO.

Avoiding damage to the deltoid muscle and minimizing the incision, especially in overweight patients, will facilitate early post-operative exercise [2–4]. Shortening the bedridden time and early exercise will decrease the incidence of complications such as DVT, pneumonia and delayed union, and help the recovery of shoulder joint function [1, 10].

Before clinical study, the attempts on NEER type IV fractures on models and animals all turned failed due to the difficulties of satisfied reduction and fixation. Therefore, in this study, we set the inclusion criteria for NEER type II and III. With the currently available surgical devices and intraoperative imaging systems, it seems impossible to implement reduction and fixation through a 5-cm incision. But we can predict that with the wide use of MIPPO technique and evolution of the surgical devices, the MIPPO technique will be applicable to NEER type IV.

## Conclusion

MIPPO through the deltoid-pectoralis approach seems superior to conventional ORIF through the deltoid-pectoralis approach in the treatment of PHF in elderly patients in terms of Constant-Murley score, NEER score, intraoperative blood loss, length of operation and SF36 score.

### Limitations

Due to the limited hospital capacity and research time, we only included 36 cases in the present study and followed them up for 4–24 months, which prevented us from obtaining absolute evidence to confirm the priority of the MIPPO technique. In addition, some older patients withdrew from the study because of severe systemic diseases, which reduced the mean age of the included patients (64.0 + 5.8 years). Therefore, more clinical trials are needed to confirm the applicability of MIPPO to patients with severe systemic diseases.
